# Effects of various sitting and standing postures on arousal and valence

**DOI:** 10.1371/journal.pone.0286720

**Published:** 2023-06-02

**Authors:** Aya Takayama, Hiroshi Sekiya

**Affiliations:** 1 Graduate School of Integrated Arts and Sciences, Hiroshima University, Hiroshima, Japan; 2 Graduate School of Humanities and Social Sciences, Hiroshima University, Hiroshima, Japan; University of Rome, ITALY

## Abstract

According to research on the effects of posture on psychological states, high-power poses—with the body spread wide open—lead to high-arousal positive emotions, whereas low-power poses—with the body slumped and constricted—lead to low-arousal negative emotions. However, postures that lead to both high-arousal negative and low-arousal positive emotions have not been investigated yet. Although relative comparisons between postures have been made, the positioning of postures on the two-dimensional coordinates created by arousal and valence has not been clarified. Therefore, the purpose of this study was to explore and clarify which postures lead to the four types of emotions: high-arousal negative, high-arousal positive, low-arousal negative, and low-arousal positive. In Experiment 1, 29 participants (13 men and 16 women) adopted 12 sitting postures for 1 minute each. In Experiment 2, 25 participants (13 men and 12 women) adopted six sitting and six standing postures for 1 minute each. Arousal and valence were measured after each posture, and heart rate was measured during posture maintenance. Arousal and valence after adopting the postures were compared with the neutral arousal and valence. As a result, postures leading to high-arousal negative and low-arousal positive emotions were identified. In addition, postures leading to high-arousal positive emotions, which are the high-power poses, were identified. There were no differences in the magnitude of psychological effects between sitting and standing postures.

## Introduction

The embodiment theory suggests that physical states, such as facial expression, posture, and movement, are related to psychological states, such as cognition and emotion [1, 2]. In particular, the idea that physical states influence psychological states is known as bodily feedback theory [3]. Studies have investigated the effects of posture, facial expression, and vocal expression on psychological states [4, 5] and concluded that manipulated bodily states influence emotional behavior, the psychophysiological processes related to emotion and motivation, and related cognitive processes. These theories are influenced by those of Darwin [6] and James [7] and are based on the James-Lange theory [7, 8], which states that bodily states cause emotions through physiological changes in the peripheral nervous system.

Research investigating the effects of bodily states on psychological states encompasses those that explore the effect on one’s own psychological state [9, 10] (intrapersonal effects) and on that of others [11] (interpersonal effects). The present study specifically focused on the intrapersonal effect, as it is consistent with both embodiment and bodily feedback theories. Thus, particular attention was given to the impact of posture on psychological states.

Riskind and Gotay [10], who first reported the effects of posture on psychological state, found that a seated upright posture increased persistence in problem-solving compared to a slumped posture, with no difference in mood or fatigue. Tilt of the head in the sitting position has also been examined, and it has been shown that an upward head tilt is associated with a positive mood, whereas a downward one is associated with a negative mood [12]. Furthermore, research has been conducted considering high-power poses as postures in which the body is spread open and back is straight and low-power poses as those in which the body is slumped and constricted. The results showed that high-power poses increased the sense of power [9, 13–16], self-esteem [13, 17], and pain tolerance [18] more than low-power poses did. In addition, some studies on power pose have used psychological questionnaires based on the two-dimensional theory of emotion [19] with arousal and valence as the two axes [13, 20]. Compared to low-power poses, high-power poses led to increased arousal levels [13] and positive emotions [13, 20] and decreased negative emotions [13, 21]. These findings suggest that high- and low-power poses lead to relatively high-arousal positive and low-arousal negative emotions, respectively.

However, considering that the two-dimensional theory of emotion includes four categories: high-arousal negative, high-arousal positive, low-arousal negative, and low-arousal positive emotions, the postures that lead to high-arousal negative and low-arousal positive emotions have not been clarified. By clarifying the postures that lead to high-arousal negative emotions (e.g., anger, tense), it may be easier to control them. In addition, McManus et al. [22] stated that high- and low-arousal positive emotions are qualitatively different. They found that mindfulness increased and anxiety and depression decreased only when low-arousal positive emotions increased. Therefore, it is necessary to clarify the postures that lead to low-arousal positive and high-arousal positive emotions. A posture that may lead to low-arousal positive emotions is that of meditation. It has been shown that the longer the meditation time, the higher the low-arousal positive emotions (e.g., calm, relaxed) [23]. Meditation involves not only posture but also other factors such as breathing and attention control. Therefore, the effect of meditation posture alone on psychological state needs to be examined.

Based on the above, it is necessary to clarify the postures that lead to high-arousal negative, high-arousal positive, low-arousal negative, and low-arousal positive emotions based on the two-dimensional theory of emotion. Although power pose studies are based on relative comparisons between high- and low-power poses, relative comparisons alone cannot clarify the applicability of the four categories indicated by the two-dimensional theory of emotion. The degree of arousal and valence in each category was not determined. Therefore, the first purpose of this study was to explore and clarify emotions induced by various postures using an index that measures subjective arousal and valence based on the two-dimensional theory of emotion.

However, relative comparisons between postures reveal changes in emotion that are not indicated by the first purpose of this study. Through relative comparisons, it is possible to determine more desirable postures and to facilitate improvements in posture. In addition, relative comparisons allow us to examine the quantitative differences in arousal and valence induced by postures in the same category. Therefore, it is important to examine the effects of posture on psychological states through relative comparisons.

Power pose studies have also examined the effects of posture on physiological status. These studies have reported that compared to low-power poses, high-power poses lead to increased testosterone levels and decreased cortisol levels [9], as well as increased pulse pressure [13]. These physiological indices measure the stress response to a task performed after posture maintenance. However, the effects of posture on the physiological state have not been investigated. Therefore, the second purpose of the present study was to relatively compare between postures in terms of their effects on psychological and physiological states.

In addition, since the James-Lange theory, on which the embodiment and bodily feedback theories are based, suggests that emotions change according to physiological changes, relationship between posture and emotion and that between physiological state and emotion during posture maintenance should be clarified. In particular, autonomic nervous system activity is related to emotions. For example, an increase in heart rate (HR) is associated with high-arousal negative (e.g., anger) and positive emotions (e.g., happiness), whereas a decrease in HR is associated with low-arousal negative (e.g., sadness) and positive emotions (e.g., contentment) [24]. Therefore, the third purpose of this study was to measure HR as an index of autonomic nervous system activity during posture maintenance and to reconfirm its relationship with subjective arousal.

Two experiments were conducted to achieve the three objectives. In Experiment 1, 12 sitting postures were compared. In Experiment 2, we used standing postures in addition to sitting postures because the psychological effects of the sitting postures were small and standing postures are expected to have large psychological effects.

## Experiment 1

### Purpose

The objectives of Experiment 1 were: 1) to explore and identify the postures that lead to high-arousal negative, high-arousal positive, low-arousal negative, and low-arousal positive emotions; 2) to determine the effects of sitting postures on arousal, valence, and HR by relatively comparing the postures; and 3) to determine the relationship between HR and subjective arousal while adopting the postures.

### Materials and methods

#### Participants

Thirty university students participated in the experiment. Statistical analysis was performed on the data of 29 participants (13 men and 16 women; *M*_*age*_ = 18.35 ± 0.56), excluding one who was aware of the cover story. The sample size was determined by a power analysis assuming a one-sample t-test. The effect size was *d* = .70, which was used by Nair et al. [13] to calculate the sample size. Twenty-nine participants were required based on the power analysis (α = .05, power = .95, two-tailed test) using G*Power3.1 [25]. Ethical considerations regarding the purpose of the experiment (cover story), method, handling of personal data, and consent for participation in the experiment were presented in writing and explained verbally before the experiment. After the experiment, a debriefing about the cover story was held, and 1,000 yen was given as an honorarium to the participants. Except one, the other 29 participants did not realize the original purpose of the experiment until the debriefing. This experiment was approved by the Research Ethics Committee of the university to which the authors belong (approval number: 30–29).

#### Postures

**Fig 1:**
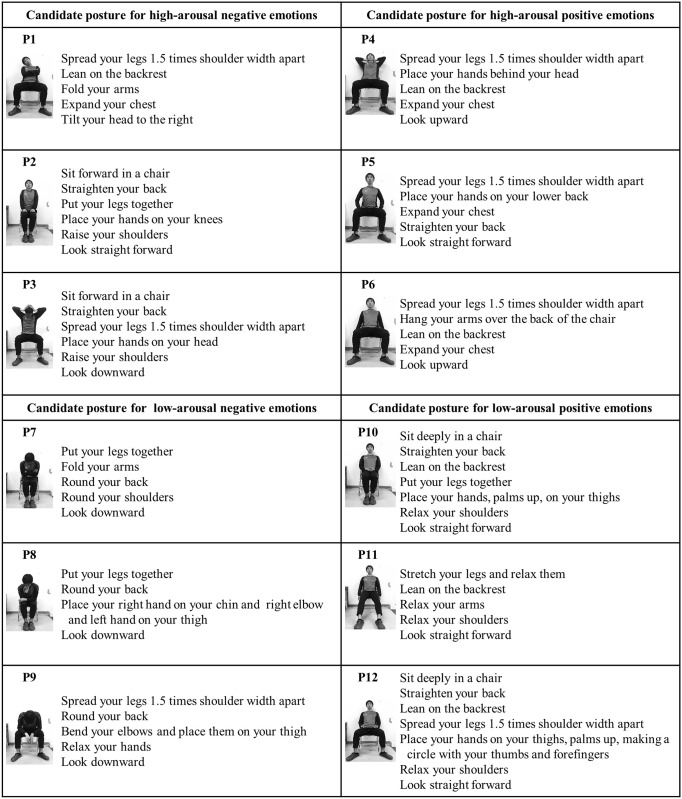
Twelve candidate postures used in Experiment 1. Twelve candidate postures were classified into the following categories based on the two-dimensional theory of emotion: high-arousal negative postures (P1–3), high-arousal positive postures (P4–6), low-arousal negative postures (P7–9), and low-arousal positive postures (P10–12).

A total of 12 sitting postures were used. Three candidate postures belonged to each of the four categories of the two-dimensional theory of emotion (Fig 1). High-arousal positive and low-arousal negative postures were selected based on a study by Carney et al. [9] that examined intrapersonal effects of high- and low-power poses. High-arousal negative and low-arousal positive postures were selected based primarily on a study by Sugimoto et al. [26] that examined intrapersonal effects and meditation postures. However, because there were few studies on the intrapersonal effects of high-arousal negative postures, we relied on a study by Wallbott [27] that examined bodily expression, specifically the use of shoulder raising as an emotional expression of “anger.” It is noteworthy that we did not use a standardized set such as the bodily expressive action stimulus test (BEAST), which is typically used in interpersonal effects studies on emotion recognition from visual stimuli, because this was an intrapersonal effects study. These postures were selected as candidates to guide each emotion and were not hypotheses to be tested. Instructions for all postures were given verbally on the basis of previous studies [9, 10]. Participants were instructed to adopt a seated upright posture in which the ear lobe, acromion, and great trochanter were in a straight line between the experimental postures. This posture was not designed to lead to the neutral emotions at the intersection of the two axes of arousal and valence, but rather to reduce the order effect of having participants adopt various postures sequentially.

#### Measures

The two-dimensional mood scale (TDMS) [28] was used to examine the psychological state during each posture maintenance. The TDMS was developed based on the two-dimensional theory of emotion and consists of eight emotion words representing the following categories: high-arousal negative (irritated, nervous), high-arousal positive (lively, energetic), low-arousal negative (lethargic, sluggish), and low-arousal positive (calm, relaxed). The questionnaire was administered using a 6-point scale ranging from 0 (*not at all*) to 5 (*extremely*). Activation (-10 to +10 points) was calculated by subtracting the total score of low-arousal negative from that of high-arousal positive, and stability (-10 to +10 points) was calculated by subtracting the total score of high-arousal negative from that of low-arousal positive. Based on these two factors, arousal (activation–stability: -20 to +20 points) and valence (activation + stability: -20 to +20 points), which are the composite indices of activation and stability, were calculated and were the subjects of analyses. The HR was measured to evaluate physiological arousal during posture maintenance. It was calculated from the R-R interval obtained from an HR monitor (RS800CX, Polar Electro Oy, Kempele, Finland) using Polar Pro Trainer 5 (Polar Electro Oy, Kempele, Finland).

#### Procedure

The experiment was conducted over a period of three days with a gap of several days. The participants were asked to adopt four different postures per day. They were told a cover story that the experiment aimed to measure their physical reactions to various postures from the medically correct posture (seated upright posture) to exclude the influence of demand characteristics, in which participants behaved according to the intention of the experimenter. Furthermore, before the experiment, they were not told that their psychological state would change based on their posture. Studies on the facial feedback hypothesis have shown that bodily state affects emotion even after the influence of demand characteristics is eliminated [29]. However, in power pose studies, it is important to reduce the influence of demand characteristics by using a cover story to divert the participants’ attention from the original purpose [30]. Considering these, to eliminate the influence of demand characteristics, the cover story for this study was set up with reference to studies [9, 10, 21, 31] that used the cover story, such as measuring physiological responses during posture holding. After the participants entered the laboratory, an HR monitor transmitter was attached to their chests for recording. First, they were asked to adopt the seated upright posture for 2 minutes, and then they responded to the TDMS to measure psychological indices during the posture. After responding, the first posture was taught verbally, and the participants were asked to adopt that posture for 1 minute. Then, they were asked to respond to the TDMS. The TDMS was repeated four times each day. The order of the 12 postures was randomized among the participants. At the end of the third day of the experiment, a debriefing was held to explain the actual research objectives and to ask whether the participants could infer what results the experimenter expected through the experiment.

#### Statistical analysis

A one-factor analysis of variance (ANOVA) was performed on each dependent variable, with experimental day (3) as a factor, to determine whether the psychological effects of the seated upright posture at the beginning of each experimental day were controlled for over a three-day period. A one-sample t-test with 0 as the criterion was performed on the arousal and valence of TDMS for each posture to compare with the neutral emotion, which is the intersection of the two axes of arousal and valence for each posture. Cohen’s *d* was used as the effect size for the t-tests. In addition, a one-sample t-test was performed on the mean values of arousal and valence in the seated upright posture measured at the beginning of all three experimental days, comparing them with the reference value of 0. Next, a multivariate analysis of variance (MANOVA) was performed to compare the relative differences among the 12 postures in the TDMS, with the arousal and valence scores of the TDMS as the dependent variables and postures as the independent variables. Pillai’s trace was used to interpret the results of the MANOVA. When Mauchly’s sphericity test could not assume equal variances in the simple main effect test, the Greenhouse-Geisser correction was used for degrees of freedom and errors. Because a significant main effect of HR was found in the one-factor ANOVA with the experimental data as a factor, an analysis of covariance (ANCOVA) was performed with HR during posture maintenance as the dependent variable, posture as the independent variable, and HR at the beginning of each experimental day as the covariate. In ANOVA and ANCOVA, *η*_*p*_^*2*^ was used for the effect size, and the Bonferroni method was used for multiple comparison tests. Finally, to examine the relationship between HR and arousal level, Pearson’s product-moment correlation analysis was performed on the mean values of HR and TDMS arousal scores for each posture.

The significance level for all tests was set at 5%. However, the one-sample t-test for the 12 postures was corrected for p-values using the Benjamini-Hochberg (BH) method [32] to reduce the increase in type I errors due to repeated statistical testing. PASW Statistics 18.0.0 software was used for the statistical analysis.

### Results and discussion

#### Manipulation check for seated upright posture

Table 1 shows the results of the seated upright posture manipulation checks. There were no significant main effects of experimental days on arousal and valence (arousal: *F* (2, 56) = 1.84, *p* = .17, *η*_*p*_^*2*^ = .06; valence: *F* (2, 56) = 1.91, *p* = .16, *η*_*p*_^*2*^ = .06). However, there was a significant main effect of experimental days on HR (*F* (2, 56) = 4.71, *p* = .01, *η*_*p*_^*2*^ = .14), and it was lower on day 1 than on days 2 and 3. The one-sample t-test on arousal and valence in the seated upright posture showed significantly smaller values for arousal (*M* = -3.15, *SD* = 4.91, *t* (86) = 5.99, *p* < .001, *d* = .64) and significantly larger values for valence (*M* = 4.69, *SD* = 3.56, *t* (86) = 12.23, *p <* .001, *d* = 1.31). This may be because the seated upright posture was described as the “medically correct posture” in the cover story, which might have induced calm feelings (low-arousal positive emotion) in the participants when they adopted the posture.

**Table 1:** Means, standard deviations, and ANOVA results for arousal and valence of TDMS and HR in the seated upright posture on each experimental day in Experiment 1.

	Day 1		Day 2		Day 3				
Variable	*M*	*SD*	*M*	*SD*	*M*	*SD*	*F*	*p*	*η*_*p*_^*2*^
Arousal	-4.00	5.43	-2.90	4.37	-2.55	4.65	1.84	.17	.06
Valence	4.48	4.02	5.38	2.64	4.21	3.75	1.91	.16	.06
HR	71.83	11.67	76.62	14.52	75.86	11.57	4.71	.01	.14

Notes: HR, heart rate; ANOVA, analysis of variance; TDMS, two-dimensional mood scale.

#### Classification of postures by comparison with neutral emotion

Table 2 presents the results of the one-sample t-test on TDMS scores for each posture. P2, 3, and 5 showed significantly higher and P1 and 6–12 showed significantly lower arousal than the criterion of 0. P5, 10, and 12 showed significantly higher and P3 showed significantly lower valence than 0. In other words, the posture with straightening the back and not leaning on the backrest led to high arousal, while that with leaning on the backrest led to low arousal. Moreover, the posture with straightening the back and looking straight forward led to positive emotions, while that with placing the hands on the head, raising the shoulder, and looking downward led to negative emotions.

**Table 2:** Means and standard deviations of TDMS arousal and valence for each posture in Experiment 1 and results of a one-sample t-test with a reference value of 0.

Variable	*M*	*SD*	*t* (28)	Adjusted *p*	Cohen’s *d*
Arousal					
P1	-4.90	6.64	3.90	.01**	0.73
P2	2.62	3.29	4.21	< .001***	0.78
P3	2.55	4.55	2.97	.02*	0.55
P4	0.38	5.62	0.36	.72	0.07
P5	2.24	3.97	2.99	.02*	0.56
P6	-3.21	6.03	2.81	.02*	0.52
P7	-8.03	5.82	7.31	< .001***	1.36
P8	-4.97	4.51	5.82	< .001***	1.08
P9	-9.28	5.36	9.17	< .001***	1.70
P10	-6.34	4.89	6.86	< .001***	1.27
P11	-12.41	3.38	19.44	< .001***	3.61
P12	-5.97	4.99	6.32	< .001***	1.17
Valence					
P1	-0.28	2.85	0.51	.64	0.10
P2	-1.03	2.67	2.05	.07	0.38
P3	-1.93	2.55	4.02	< .001***	0.75
P4	0.86	4.39	1.04	.34	0.19
P5	3.28	3.80	4.56	< .001***	0.85
P6	-1.21	3.03	2.11	.07	0.39
P7	-0.93	2.92	1.69	.13	0.31
P8	-1.03	3.30	1.66	.12	0.31
P9	-1.14	3.09	1.95	.08	0.36
P10	3.31	3.30	5.31	< .001***	0.99
P11	1.17	3.71	1.67	.13	0.31
P12	4.03	3.61	5.91	< .001***	1.10

Adjusted *p*-value indicates the p-value corrected using the Benjamini-Hochberg (BH) method.

* denotes *p* < .05,

** denotes *p* < .01,

*** denotes *p* < .001.

TDMS, Two-dimensional mood scale.

P2, 3, and 5, which led to high arousal, had the instruction “straighten your back” based on the previous finding that compared to low-power poses, straight postures (high-power poses) increase more high-arousal positive emotions [13]. The findings revealed that a straight posture without leaning on the backrest led to higher arousal. In addition, the candidate postures that led to low arousal (P7–12) led to significantly lower arousal. These postures were set based on the fact that compared with high-power poses, constricted postures (low-power pose) increase more low-arousal negative emotions [8], and that supporting the body with a backrest may lead to feelings of relaxation (low-arousal positive emotion). Therefore, the candidate postures with the instruction “lean on the backrest” that led to high arousal, also led to low arousal. These results indicate that the postures referring to the low-power pose (P7–9) and that of leaning on the backrest led to lower arousal compared to the neutral emotion. P1 and 6, which did not lead to high arousal, included a “lean on the backrest” instruction, which was not present in postures that led to high arousal. This may have prevented these postures from leading to high arousal. This instruction was used in P1 and 6, which were high arousal candidates, to maintain the postures in which the body was spread wide, which is a characteristic of high-power poses. However, this instruction was also used in the candidates P10–12, which led to low-arousal positive emotions because support by the backrest to the body was led to feelings of relaxation (low-arousal positive emotion). The present results suggest that the “lean on the backrest” instruction had a greater effect on supporting the body than on making the body appear larger, leading to feelings of relaxation.

Regarding valence, P5, 10, and 12 were significantly positive among the candidate postures that led to positive emotions (P4–6 and P10–12), and only P3 was significantly negative among the candidate postures that led to negative emotions (P1–3 and P7–9). On the contrary, P1, 2, 4, 6, 7–9, and 11 led to neutral feelings in terms of valence. P5, 10, and 12, which led to positive emotions, differed from other candidate postures that led to positive emotions in that they had both instructions of “straighten your back” and “look straight forward.” In other words, the instructions “straighten your back” and “look straight forward” together led to positive emotions. However, P2, which led to negative emotions as a candidate, did not lead to positive emotions, even though both instructions were included. The reason for this may be that P2 was set up as a freezing posture. Freezing is an adaptive behavior that occurs when an individual faces a threat; its relationship with increased muscle activity has been demonstrated [33]. Therefore, a posture in which the upper body is tense may express a state of freezing, leading to negative emotions. However, it is possible that the positive emotions evoked by “straighten your back” and “look straight forward” were offset by the negative emotions evoked by the freezing posture. Only P3, which led to high-arousal negative emotions as a candidate with reference to the report that shoulders rise when feeling anger [27] and head is held in hands when feeling anguish, led to negative emotions. Moreover, P3 had the instruction to “look downward” because a downward-facing head is associated with negative emotions [12]. Therefore, it can be considered that the postures of placing hands on head, raising shoulders, and looking downward led to negative emotions.

**Fig 2:**
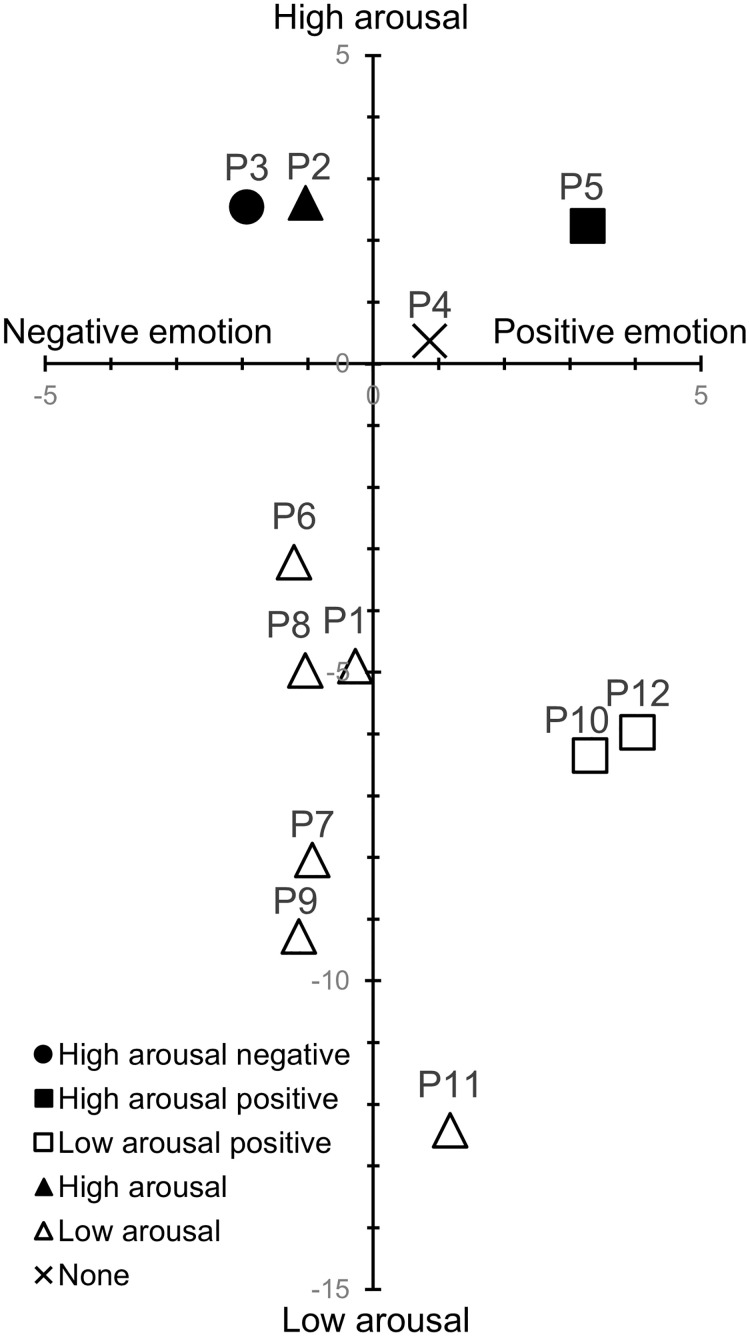
2-D plot of TDMS arousal and valence for each posture in Experiment 1. The markers reflect the results of a one-sample t-test. Filled and white markers indicate the postures that led to high and low arousal, respectively. Square and circle represent the postures that led to positive and negative emotions, respectively. Triangles indicate postures that had an effect only on arousal, and cross marks indicate postures that had no effect on both arousal and valence.

Classifying these results based on the two-dimensional theory of emotion, we found that P3 led to high-arousal negative emotions; P5, high-arousal positive emotions; and P10 and 12, low-arousal positive emotions (Fig 2). P3, which led to high-arousal negative emotions, included not leaning on the backrest, placing hands on the head, raising shoulders, and looking downward. P5, with hands placed on the lower back and expanded chest, was based on reports that compared with low-power poses, high-power ones enhance more high-arousal positive emotions [13], and it led to high-arousal positive emotions as a candidate. This experiment found that a high-power pose, in which the participants placed their hands on their lower back and expanded their chest, led to higher arousal of positive emotions than neutral ones. P10 and 12 referred to meditation postures and led to low-arousal positive emotions, as candidates. It has been shown that low-arousal positive emotions increase as meditation duration increases [23]. In this experiment, it was shown that adopting the meditation posture for 1 minute, without actually meditating, led to low-arousal positive emotions.

None of the postures in this experiment were classified as leading to low-arousal negative emotions. The candidate postures that led to low-arousal negative emotions (P7–9) led to low arousal, but not negative emotions in the participants. The TDMS measures low-arousal negative emotions with the expressions “lethargic” and “sluggish.” However, if P7–9 caused other negative emotions that did not correspond to these expressions, they might not be reflected as negative emotions in the TDMS. In a previous study, participants in low-power poses reported emotional words, such as sleepy and tired [34]. Emotions such as sleep and tiredness are positioned as low arousal in a circular model of affect [19]. P7–9, include forward-leaning, therefore, it may have led to low arousal such as sleep and tiredness instead of lethargy and sluggishness, which are not reflected as negative emotions in the TDMS.

#### Comparison between 12 different postures

A MANOVA of posture (12) was performed on the arousal and valence scores of the TDMS. Posture had a significant main effect (*F* (11, 18) = 16.77, *p* < .001, *η*_*p*_^*2*^ = .91). One-way ANOVA was performed to examine the simple main effect of each TDMS score. Significant main effects were observed for arousal and valence (arousal: *F* (6.57, 34.15) = 35.09, *p* < .001, *η*_*p*_^*2*^ = .56; valence: *F* (5.85, 17.83) = 13.21, *p* < .001, *η*_*p*_^*2*^ = .32). The multiple comparison test showed that, P2, 3, and 5 had higher arousal scores than P1 and 6–12 (*p*s < .05), and P4 had higher scores than P7 and 9–12 (*p*s < .05). Moreover, P6 and 8 had higher scores than P9 and 11 (*p*s < .05), and P1, 7, 10, and 12 had higher scores than P11 (*p*s < .05). This shows that postures with high physical burden, such as straightening the back, raising the shoulders, and expanding the chest, were significantly more arousing than those with low physical burden, such as rounding the back and leaning on the backrest. Even among the postures that showed low arousal in the one-sample t-test, differences were observed depending on the physical burden. The valence scores of P5 and 10 were higher than those of P1–3 and 6–9 (*p*s < .05); P11 was higher than that of P3 (*p* < .05); and P12 was higher than that of P1–4 and 6–9 (*p*s < .05). Put differently, the postures that involved placing the hands on the lower back and expanding the chest and the meditation posture led to more positive emotions.

In terms of arousal, P2, 3, and 5 were more arousing than other postures. These postures had instructions to not only “straighten your back” but also to “raise your shoulders” or “expand your chest,” increasing the physical burden. The postures that showed significantly higher arousal were those that involved less physical burden, such as rounding the back or leaning against the backrest. Studies have shown that the high-power pose of straightening the back leads to higher arousal than the low-power pose of rounding the back [13]. Experiment 1 showed that not only the postures with straightening the back, but also those with raising the shoulder and expanding the chest led to higher arousal than those with rounding the back and leaning on the backrest. P9 and 11 showed lower arousal than P6 and 8, indicating that the degree of low arousal differed among the postures. P6 and 11, as well as P8 and 9, showed differences in arousal levels, although their postures were quite similar. P6 and 8 had the instructions to “expand your chest” and “place your right hand on your chin and right elbow, and your left hand on your thigh,” respectively. Conversely, P9 and 11 had the instructions to “relax your hands” and “relax your arms,” respectively, which may have resulted in lesser physical burden. This difference in the degree of physical burden may have influenced the difference in the degree of low arousal.

Next, P5, 10, and 12 showed higher levels of valence than most of postures. These postures included placing the hands on the lower back and expanding the chest, which is based on the high-power pose and the meditation posture. The postures with tilting the head to the right, raising the shoulder and rounding the back, or looking downward showed lower levels of valence than these postures. In Experiment 1, the posture involving placing the hands on the lower back and expanding the chest showed a higher level of valence than those with rounding the back or looking downward. These results support the reports that high-power poses lead to more positive emotions than low-power poses [13, 20, 21]. Furthermore, the meditation posture showed a higher level of valence than the postures with tilting the head to the right, raising the shoulder and rounding the back or looking downward.

**Fig 3:**
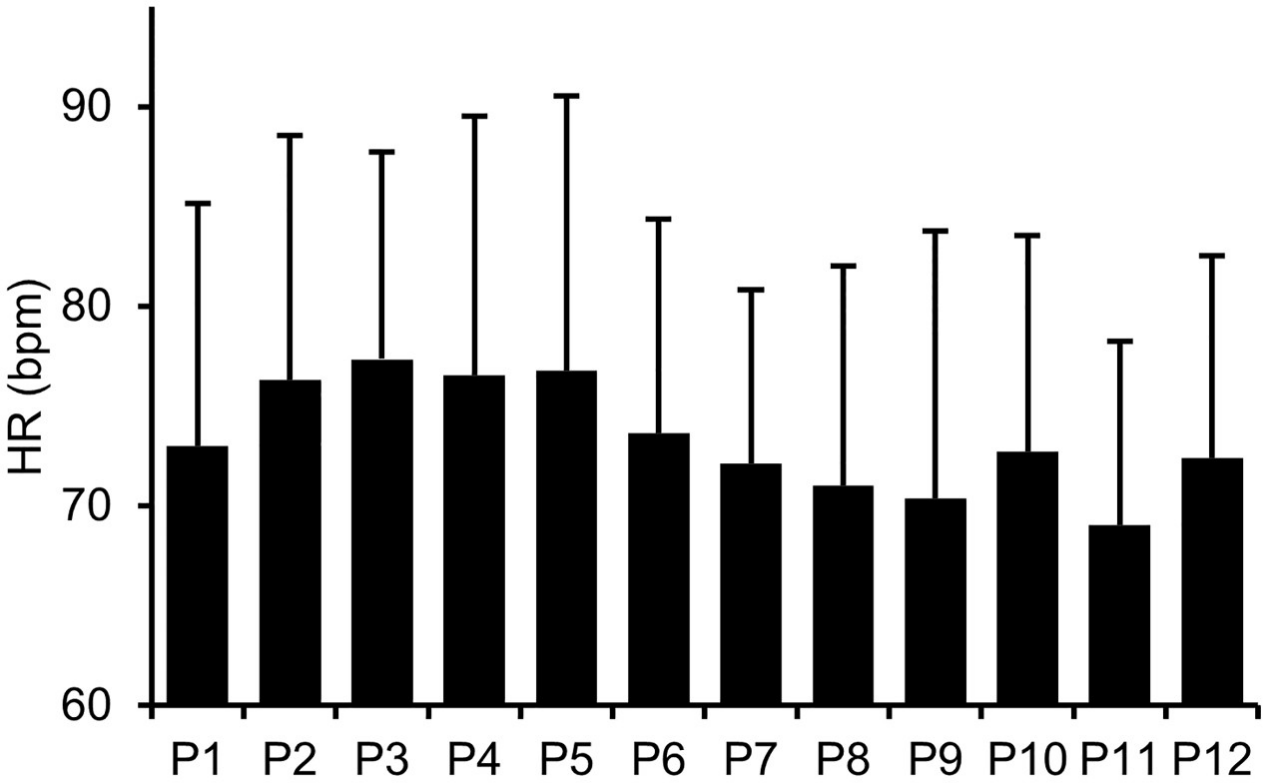
HR for each posture in Experiment 1. Error bars indicate standard deviations.

The HR for each posture is shown in Fig 3. The ANCOVA revealed a significant main effect (*F* (11, 335) = 3.14, *p* < .001, *η*_*p*_^*2*^ = .09). The multiple comparison test showed that HR was higher for P3 than for P9 and 11 (*p*s < .05) and for P5 than for P11 (*p*s < .05), indicating that P3 and 5 were postures with relatively high physical strain, such as not leaning on the backrest, placing hands on the head, raising shoulders, or placing hands on the lower back and expanding the chest. Whereas P9 and 11 were postures with low physical strain. This difference in physical burden may have affected the magnitude of the HR. However, the sitting postures used in this experiment, which included instructions to increase upper body muscle activity leading to high arousal, did not show as many significant differences as the results of the comparison between postures in the TDMS arousal level. To highlight more differences between postures at the physiological arousal level, it may be effective to include standing posture [35], which have higher muscle activity and an increased HR than sitting postures have.

#### Relationship between HR and TDMS arousal

Pearson’s product-moment correlation analysis of HR for each posture and the mean subjective arousal measured by TDMS showed a significantly high positive correlation (*r* = .96, *p* < .001), supporting the report that HR and subjective arousal are related [24]. In conjunction with the relative comparisons between the sitting postures, both objective and subjective arousal levels were expected to increase when the participants adopted postures with high physical burden. In contrast, both objective and subjective arousal levels were expected to decrease when they adopted postures with low physical burden. Moreover, the results of the relative comparisons of HR and subjective arousal between postures were generally consistent, indicating that HR and subjective arousal were related in this experiment. Furthermore, although a strong correlation was found between the objective and subjective measures of arousal, more significant differences between postures were found in the subjective measure of arousal in the TDMS, indicating that it had a higher discriminative power.

## Experiment 2

### Purpose

Experiment 1 revealed postures that lead to high-arousal negative, high-arousal positive, and low-arousal positive emotions. Although the possible scores for arousal and valence ranged from -20 to +20 points, the observed valence scores ranged from -1.93 (P3) to +4.03 (P12) points, indicating a small range. The highest arousal score was +2.62 (P2). Therefore, we considered standing postures to increase the effects of subjective arousal and valence. It has been shown that work in the standing posture has higher arousal and lower valence than that in the sitting posture [36]. Moreover, work that includes standing posture leads to more positive feelings than that which includes only sitting posture [37]. In addition, the standing posture has a higher HR than the sitting posture [35], and an increase in the level of physiological arousal. Therefore, in Experiment 2, some of the sitting postures in Experiment 1 were replaced by standing postures and the same objectives were examined.

### Materials and methods

#### Participants

Twenty-five university students (13 men, 12 women; *M*_*age*_ = 20.32 ± 1.95) who did not participate in Experiment 1 were involved in Experiment 2. As in Experiment 1, the sample size was determined by a power analysis assuming a one-sample t-test. The effect size was *d* = .99, which was the median effect size of the significant one-sample t-test results in Experiment 1. Sixteen participants were required based on the power analysis (α = .05, power = .95, two-tailed test) using G*Power3.1 [25]. In the post-experiment debriefing, it was confirmed that none of the participants recognized the original purpose of the experiment and that they were not aware of it until the debriefing. Ethical considerations and honorarium were the same as in Experiment 1 (approval number: 01–57).

#### Postures

**Fig 4:**
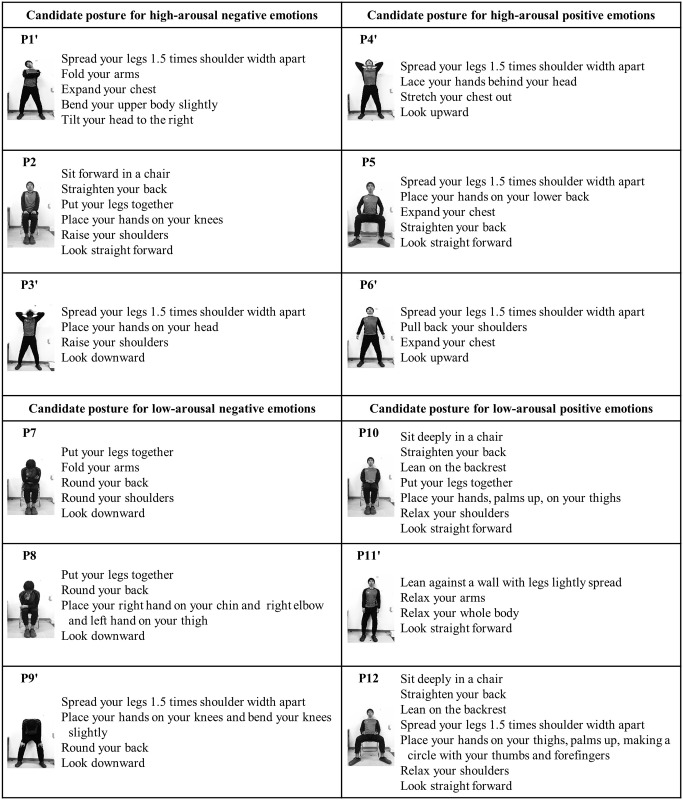
Twelve candidate postures used in Experiment 2. Twelve candidate postures were classified into the following categories based on the two-dimensional theory of emotion: high-arousal negative postures (P1’, 2, and 3’), high-arousal positive postures (P4’, 5, and 6’), low-arousal negative postures (P7, 8, and 9’), and low-arousal positive postures (P10, 11’, and 12). Standing postures are indicated by the prime symbol.

Twelve postures (Fig 4) were used in this experiment. Two of the three candidate postures that led to the high-arousal negative and positive emotions and were used in Experiment 1, were changed to standing postures. One of the three candidate postures that led to low-arousal negative and positive emotions and were used in Experiment 1, was changed to a standing posture. Therefore, P1, 3, 4, 6, 9, and 11 were changed to standing postures (P1’, 3’, 4’, 6’, 9’, and 11’) in Experiment 2. The other postures were identical to the ones used in Experiment 1. The reason for including both standing and sitting postures was to examine the differences between them within the same category. Between trials, the same seated upright posture as in Experiment 1 was used. Each participant had to participate in the experiment for two days, which was different from Experiment 1. Therefore, we were unable to compare the two experiments. We included standing postures in all categories to compare their effects only within Experiment 2.

#### Measures

The TDMS was used to examine the psychological state during the first seated upright posture maintenance on each experimental day and during each posture maintenance, as in Experiment 1, and arousal and valence were calculated. The HR was measured to evaluate physiological arousal during posture maintenance. It was calculated from the R-R interval obtained from an HR monitor (V800, Polar Electro Oy, Kempele, Finland) using Kubios HRV software version 3.1 (Kubios Oy, Kuopio, Finland [38]).

#### Procedure

The experiment was conducted over a period of two days with a gap of several days, and the participants were asked to adopt six different postures per day. In Experiment 1, the seated upright posture induced by the cover story might have led to low-arousal positive emotions. Therefore, we omitted the phrase “medically correct posture” in Experiment 2 and used a cover story that the experiment was to measure their physical reactions to various postures. The procedure for each trial was the same as in Experiment 1, and six trials were repeated per day in Experiment 2. At the end of the second day, a debriefing was held to explain the true research objectives and to ask the participants whether they had inferred the experimenter’s expectations about the results.

#### Statistical analysis

A paired t-test was performed on each dependent variable to determine whether the psychological effects of the seated upright posture at the beginning of each experimental day were controlled for over a two-day period. Other statistical analyses were conducted similarly as in Experiment 1.

### Results and discussion

#### Manipulation check for seated upright posture

Table 3 shows the results of the seated upright posture manipulation checks. There were no significant differences between the experimental days for all measurements (arousal: *t* (24) = 1.12, *p* = .28. *d* = 0.22; valence: *t* (24) = 0.42, *p* =. 68, *d* = .08; HR: *t* (24) = 0.15, *p* =. 88, *d* = 0.31), indicating that the seated upright posture at the beginning of each experimental day was controlled for over two days. The one-sample t-test on arousal and valence in the seated upright posture showed significantly smaller and larger values than the criterion of 0 for arousal (*M* = -4.78, *SD* = 4.99, *t* (49) = 6.77, *p* < .001, *d* = 0.96) and valence (*M* = 4.90, *SD* = 4.41, *t* (49) = 7.86, *p* < .001, *d* = 1.11), respectively, despite the exclusion of the phrase “medically correct posture” in the cover story. Therefore, the seated upright posture leads to low-arousal positive emotions regardless of usage of the phrase “medically correct posture.”

**Table 3:** Means, standard deviations, and paired t-test results for arousal and valence of TDMS and HR in the seated upright posture for each experimental day in Experiment 2.

	Day 1		Day 2				
Variable	*M*	*SD*	*M*	*SD*	*t* (24)	*p*	Cohen’s *d*
Arousal	-5.89	5.29	-4.28	4.51	1.12	.28	0.22
Valence	5.08	3.40	4.72	5.15	0.42	.68	0.08
HR	76.91	9.88	77.58	13.31	0.15	.88	0.31

Note: HR, heart rate; TDMS; two-dimensional mood scale.

#### Classification of postures by comparison with neutral emotion

Table 4 presents the results of the one-sample t-test on the TDMS scores for each posture. In terms of arousal, no posture was significantly higher than the criterion of 0, and P7, 8, 10, 11’, and 12 were significantly low. In terms of valence, P4’, 5, 6’, 10, and 12 were significantly higher than 0, and no posture was significantly low. The sitting posture with rounding the back and looking downward, sitting meditation posture, and standing posture with the whole body relaxed led to low arousal. Furthermore, the sitting meditation posture and sitting and standing postures with expanding the chest and looking straight forward or upward led to positive emotions.

**Table 4:** Means and standard deviations of TDMS arousal and valence for each posture in Experiment 2, and results of a one-sample t-test with a reference value of 0.

Variable	*M*	*SD*	*t* (28)	Adjusted *p*	Cohen’s *d*
Arousal					
P1’	0.68	5.86	0.57	.69	0.11
P2	1.68	4.51	1.83	.14	0.37
P3’	2.28	5.33	2.09	.09	0.42
P4’	0.80	5.05	0.78	.63	0.16
P5	0.64	4.44	0.71	.65	0.14
P6’	2.68	5.75	2.29	.06	0.46
P7	-5.92	4.72	6.14	< .001***	1.23
P8	-7.24	5.43	6.53	< .001***	1.31
P9’	-0.72	5.10	0.69	.63	0.14
P10	-6.76	6.45	5.14	< .001***	1.03
P11’	-7.12	5.87	5.94	< .001***	1.19
P12	-6.72	5.34	6.16	< .001***	1.23
Valence					
P1’	0.20	4.30	0.23	.85	0.05
P2	-0.80	3.57	1.12	.44	0.22
P3’	-0.52	3.28	0.79	.65	0.16
P4’	3.28	5.47	3.00	.02*	0.60
P5	2.88	4.69	3.07	.02*	0.61
P6’	2.92	3.72	3.93	.002**	0.79
P7	-0.40	3.69	0.54	.68	0.11
P8	0.04	3.05	0.07	.95	0.01
P9’	-0.40	4.14	0.48	.69	0.10
P10	2.92	3.65	4.00	.002**	0.80
P11’	1.68	3.58	2.35	.06	0.47
P12	4.24	3.72	5.70	< .001***	1.14

Adjusted *p* indicates the p-value corrected using the BH method.

* denotes *p* < .05,

** denotes *p* < .01,

**** denotes *p* < .001.

TDMS, two-dimensional mood scale.

The candidate postures that led to high arousal (P1’, 2, 3’, 4’, 5, and 6’) did not actually lead to high arousal. The candidate postures that led to low arousal (P7, 8, 9’, 10, 11’, and 12) actually led to low arousal, except for P9’. A previous study reports that a low-power pose with a rounded back shows lower arousal compared to high-power poses [13]. In this study, however, rounding the back and facing down posture led to lower arousal even compared to a neutral emotion. Additionally, as in Experiment 1, the meditation postures of P10 and 12 led to low arousal. Furthermore, P11’, despite being a standing posture, led to low arousal. This posture involves leaning against a wall and relaxing the whole body, resulting in little physical burden despite being a standing posture. Therefore, it is considered that the subjective arousal felt was low, leading to low arousal. We adopted standing postures based on a previous study showing that work in the standing posture leads to higher arousal than that in the sitting posture [37]. However, the standing postures which led to high arousal as candidates (P1’, 3’, 4’, and 6’) did not lead to high arousal in Experiment 2. The previous study compared the relative arousal of standing and sitting postures, and the posture maintenance time was 30 minutes, which was much longer than the 1 minute duration in the present experiment. The extent to which standing posture maintenance time influences higher than neutral arousal needs to be investigated in future research.

The candidate postures that led to positive emotions (P4’, 5, 6’, 10, 11’, and 12) led to positive emotions in the experiment, except for P11’. There are reports that high-power poses lead to more positive emotions compared to low-power poses [13, 20]. However, in this experiment, high-power poses with expanding the chest and looking straight forward or upward led to positive emotions rather than neutral emotions. Additionally, as in Experiment 1, meditation postures (P10 and 12) induced positive emotions. All candidate postures that led to negative emotions (P1’, 2, 3’, 7, 8, and 9’) did not lead to negative emotions in the experiment. The sitting (P2, 7, and 8) and standing (P1’, 3’, and 9’) postures that led to negative emotions as candidates did not lead to negative emotions, as in Experiment 1.

**Fig 5:**
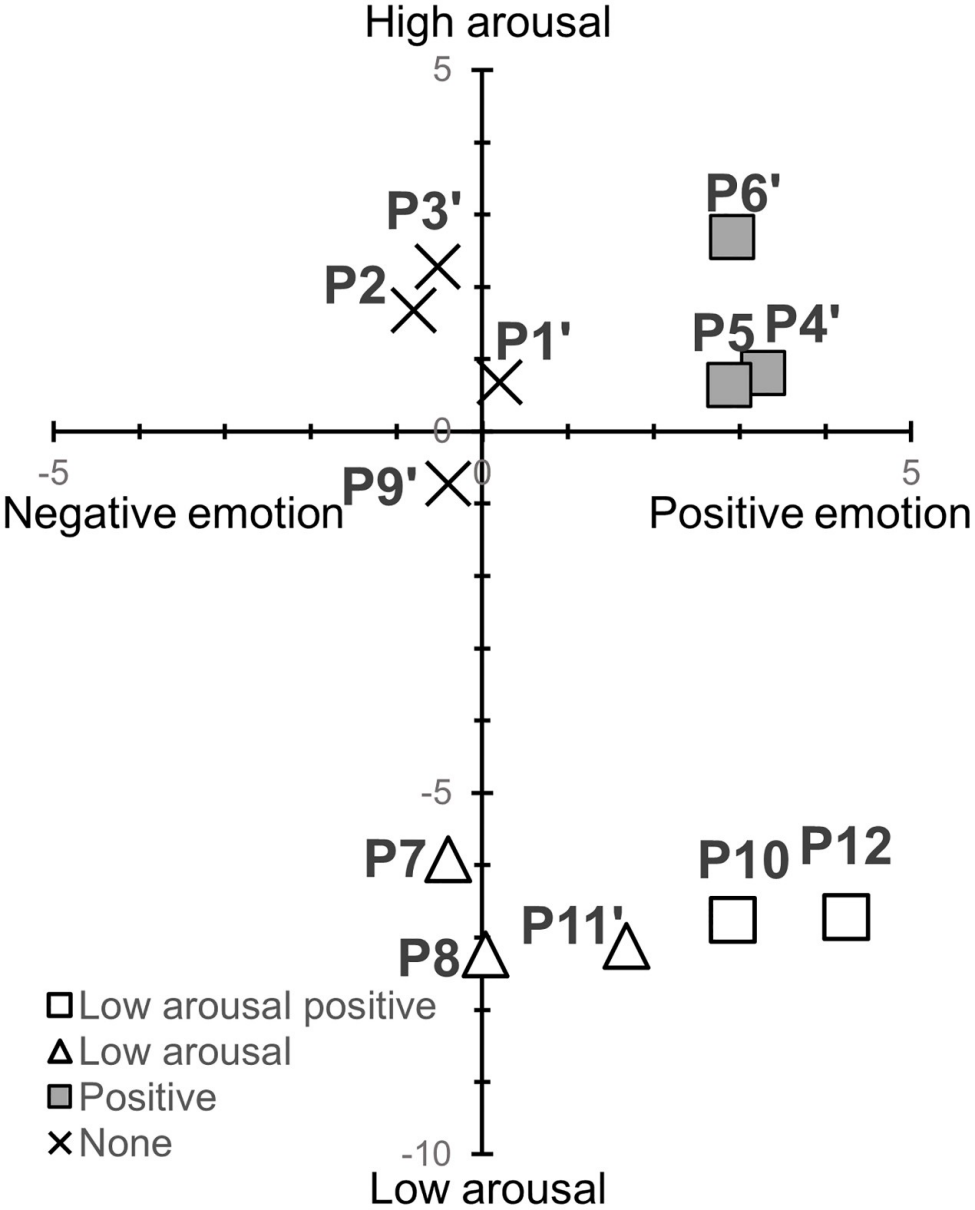
2-D plot of TDMS arousal and valence for each posture in Experiment 2. The markers reflect the results of the one-sample t-test. White and gray markers indicate postures that led to low arousal and those that had no effect on arousal, respectively. The square and cross markers indicate the postures that led to positive emotions and those that had no effect on either arousal or valence.

The only postures with a significant difference in both arousal and valence compared to neutral emotion were P10 and 12, categorized as low-arousal positive emotions (Fig 5). No postures were identified as high-arousal negative or positive, as in Experiment 1, because none led to a significantly higher arousal in Experiment 2. In addition, no postures were identified as high-arousal negative or low-arousal negative because none led to significantly negative emotions in Experiment 2.

#### Comparison between 12 different postures

A MANOVA of posture (12) was conducted on the arousal and valence scores of the TDMS. There was a significant main effect of posture (*F* (11, 14) = 5.97, *p* = .001, *η*_*p*_^*2*^ = .82). A one-way ANOVA was performed to examine the main effect of each TDMS score. A significant main effect was revealed in both arousal and valence (arousal: *F* (5.85, 140.51) = 816.13, *p* < .001, *η*_*p*_^*2*^ = .50; valence: *F* (5.78, 158.98) = 6.86, *p* < .001, *η*_*p*_^*2*^ = .22). The multiple comparison test showed that, P1’, 2, 3’, 4’, 5, 6’, and 9’ had higher arousal scores than P7, 8, 10, 11’, and 12 (*p*s < .05). In other words, as in Experiment 1, postures with high physical burden, such as straightening the back, raising the shoulders, and expanding the chest, were significantly more arousing than those with low physical burden, such as rounding the back and leaning on the backrest. The valence scores of P5 and 6’ were higher than those of P2 and 3’ (*p*s < .05), P10 was higher than that of P2 (*p* < .05), and P12 was higher than those of P1’, 2, 3’, 7, 8, and 9’ (*p*s *<* .05). This means that postures involving placing the hands on the lower back and expanding the chest, expanding the chest and looking upward, and meditation led to more positive emotions than those with raising the shoulder and placing the hands on the head.

In terms of arousal, as in Experiment 1, the results showed that not only the postures with straightening the back, but also those with raising the shoulder and expanding the chest led to higher arousal than those with rounding the back and leaning on the backrest. The standing low-power pose (P9’) was not significantly different from the high-power poses because of the half-sitting posture, which has a physical burden on the legs and arms.

In terms of valence, the postures that involved placing the hands on the lower back and expanding the chest or expanding the chest and looking upward, which were based on high-power poses, led to more positive emotions than did those with raising the shoulder and placing the hands on the head. High-power poses have been reported to increase positive emotions more than low-power poses [13, 20]. In the present study, it was further revealed that these postures led to higher positive emotions than those that involve raising the shoulder and placing the hands on the head. Furthermore, the meditation posture led to more positive emotions than many other postures. Studies have reported higher subjective energy (“in control,” “powerful,” “energetic,” and “empowered”) in yoga poses than in high-and low-power poses [39]. Items such as “powerful” and “energized” are high-arousal positive emotion words, suggesting that the meditation postures, which are also used in yoga, might have led to more positive emotions than other postures.

**Fig 6:**
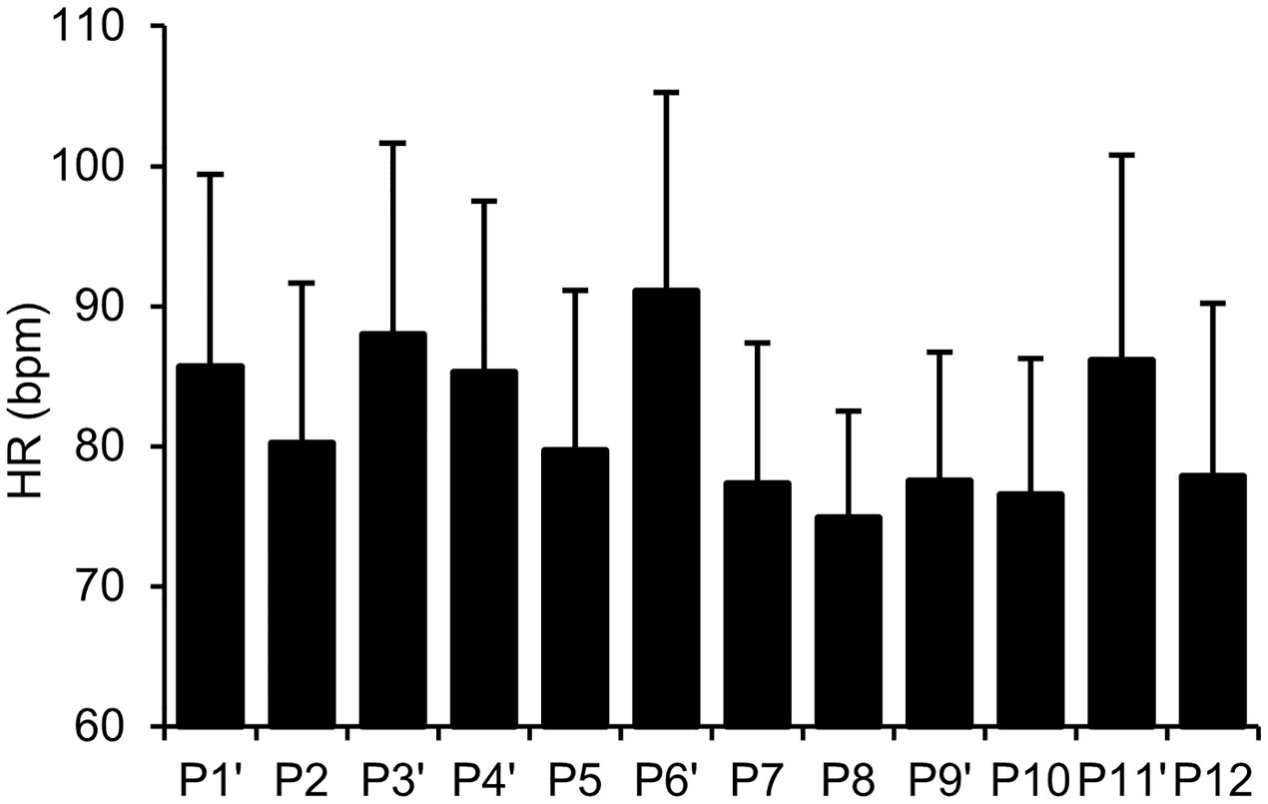
HR for each posture in Experiment 2. Error bars indicate standard deviations.

The HR for each posture is shown in Fig 6. ANOVA revealed a significant main effect (*F* (6.13, 146.94) = 13.30, *p* < .001, *η*_*p*_^*2*^ = .36). The multiple comparison test showed that the HR was higher for P1’ than for P8, 10, and 12 (*p*s < .05); for P3’ and 6’ than for P2, 5, 7, 9’, 10, and 12 (*p*s < .05); for P4’ than for P8 (*p* < .05); and for P11’ than for P8, 10, and 12 (*p*s < .05). The postures that showed significantly higher HR were the standing ones, supporting the report that standing ones show higher HR than sitting postures owing to higher muscle activity [35].

#### Relationship between HR and TDMS arousal

Pearson’s product-moment correlation analysis of the HR for each posture and mean subjective arousal measured by the TDMS showed a moderately positive correlation (*r* = .61, *p* < .04). This result supports the report that HR and subjective arousal are related [24]. In conjunction with the relative comparisons between postures, both objective and subjective arousal levels are expected to increase when people adopt upright or standing postures. In addition, the results of the relative comparisons of HR and subjective arousal between postures were generally consistent, indicating that HR and subjective arousal were related in this experiment.

## General discussion

The most important purpose of this study was to explore and clarify the postures that lead to high-arousal negative, high-arousal positive, low-arousal negative, and low-arousal positive emotions based on the two-dimensional theory of emotion. Classification of the postures based on the combination of arousal and valence showed that P3 and 5 led to high-arousal negative and positive emotions, respectively, in Experiment 1. P10 and 12 led to low-arousal positive emotions in both Experiments 1 and 2.

P10 and 12 are postures used in meditation, and this study revealed that the postures alone lead to low-arousal positive emotions without the breathing and attention control required for meditation. McManus et al. [22], who suggested the importance of low-arousal positive emotions, stated that the reason for measuring only high-, not low-, arousal positive emotions in previous studies was that unlike low-arousal positive emotions, high-arousal ones were easy to distinguish from other positive emotions. In the present study, we identified the postures that lead to high- and low-arousal positive emotions using a questionnaire that distinctly measured each emotion.

Experiment 1 showed that P3 led to high-arousal negative emotions, a category not considered in power pose studies. However, when P3 was changed to the standing posture (P3’) in Experiment 2, it led to neither subjective high arousal nor negative emotions. Although a previous study showed that prolonged standing decreased comfort more than prolonged sitting [36]. It is possible that the short duration of posture maintenance (1 minute) in the present study did not lead to negative emotions. It is also possible that the participants felt less discomfort in P3’, in which the legs were extended, compared to P3, in which both legs and arms were flexed.

P5, which led to high-arousal positive emotions in Experiment 1, did not lead to high arousal but to positive emotions in Experiment 2. This posture, which includes both straightening the back and looking straight forward, led to positive emotions. The reason for this posture not leading to high arousal in Experiment 2 may be that the subjective arousal of P5, the sitting posture, was underestimated because many standing postures were used in Experiment 2. Throughout Experiments 1 and 2, the mean HR in the sitting posture was less than 80 bpm in all postures, whereas that in the standing postures in Experiment 2 was above 80 bpm, except for P9’. Although physiological arousal increased when the sitting postures in Experiment 1 were changed to standing postures in Experiment 2, the subjective arousal in the sitting postures was estimated to be lower than that in the standing postures in Experiment 2.

Relative comparisons between postures revealed more information than that found by previous power pose studies, such as high-power poses lead to increased arousal [13], increased positive emotions [13, 20], and decreased negative emotions [13, 21] compared to low-power poses. For example, postures with high physical burden showed higher arousal than those with low physical burden. Postures that required placing the hands on the lower back and expanding the chest or expanding the chest and looking upward led to more positive emotions than did those with raising the shoulder and placing the hands on the lower back. Furthermore, the meditation posture showed a higher valence than the high-power poses. These results suggest that if someone wants to control their level of arousal, they should pay attention to their physical burden. If an individual uses postures such as rounding their back, looking downward, raising their shoulders, and placing their hands on their head, they can improve their valence by straightening their back, placing their hands on their lower back, expanding their chest, or looking straight forward or upward. Therefore, changing posture is expected to improve one’s mood.

With regard to the third purpose, the relationship between HR and TDMS arousal was highly and moderately correlated in Experiments 1 and 2, respectively, supporting previous research showing that HR was related to subjective arousal [24]. Therefore, we can control arousal by adopting postures that increase HR (e.g., standing posture, expanding chest posture, or raising shoulder posture) to increase subjective arousal and postures that decrease HR (leaning on the backrest or rounding back) to decrease subjective arousal.

In Experiment 2, we assumed that changing some of the sitting postures in Experiment 1 to standing ones would have a pronounced effect on arousal and valence; however, this was not observed. Conversely, even when it is difficult to adopt a standing posture, it is possible to change arousal and valence by using sitting postures. In addition, no significant low-arousal negative postures were identified in the two experiments. In the other three categories, it should be further examined whether postures other than those used in this study and physical conditions other than postures can produce more significant psychological effects. For example, it has been reported that slouched walking decreases psychological arousal compared to skipping [40] and leads to low-arousal negative emotions compared to upright walking [41]. Thus, psychological effects of a combination of posture and movement are worth examining.

To identify more postures that lead to significant arousal and valence, it may be necessary to use questionnaires other than the TDMS that measure subjective arousal and valence. If the TDMS is used, emotions other than those corresponding to the questions included in it may not be reflected in the scores, even if they are derived. For example, in the TDMS, negative emotions are measured by only four items: “irritated,” “nervous,” “lethargic,” and “sluggish,” however, other negative emotions such as sadness, disgust, and fear cannot be measured. A possible solution to this is the Affect Grid [42], in which participants can directly answer arousal and valence questions using two-dimensional coordinates with arousal and valence as the two axes. Although the Affect Grid can measure arousal and valence, it cannot measure the quality of the generated emotion. Hence, when using the Affect Grid, it is necessary to investigate emotions during posture maintenance using an open-ended question after posture maintenance. Future work is required to examine the effects of posture and movement on arousal and valence using these methods.
